# Relationship between Screening, Diagnostic Mammograms, Hospital Admissions, and Mortality Rates from Breast Cancer

**DOI:** 10.3390/ijerph21081006

**Published:** 2024-07-31

**Authors:** Kely Paviani Stevanato, Helena Fiats Ribeiro, Lander dos Santos, Fernando Castilho Pelloso, Pedro Beraldo Borba, Deise Helena Pelloso Borghesan, Maria Dalva de Barros Carvalho, Raíssa Bocchi Pedroso, Constanza Pujals, Sandra Marisa Pelloso

**Affiliations:** 1 Health Sciences Center, State University of Maringá-UEM, Maringá 87020-900, Brazil; helenafiats@hotmail.com (H.F.R.); lander_ds@hotmail.com (L.d.S.); mdbcarvalho@gmail.com (M.D.d.B.C.); raissap@gmail.com (R.B.P.); constanza.pujals@gmail.com (C.P.); smpelloso@gmail.com (S.M.P.); 2 Secretary Health Curitiba, Curitiba 80060-240, Brazil; fercaspell@ufpr.br; 3 Department of Medicine, University of Marilia-UNIMAR, Marilia 17525-902, Brazil; bborbapedro@gmail.com; 4 Department of Aesthetics and Cosmetics, Catholic College of Mato Grosso, Várzea Grande 78070-200, Brazil; prof.deisepelloso@uninga.edu.br

**Keywords:** breast cancer, diagnosis, hospitalization, death, tracking

## Abstract

Background: Breast cancer is the most common type of cancer worldwide. If diagnosed and treated early, it has a high chance of cure, and for this, screening tests are necessary, namely mammograms, which are the most commonly used. The objective of this study was to analyze the association between the number of screening and diagnostic mammograms and the number of hospitalizations and deaths from breast cancer. Methods: This is a cross-sectional, analytical, retrospective study with secondary data made available by the Ministry of Health. Pearson correlation analysis was employed to assess whether the number of mammograms is associated with the number of deaths and hospitalizations, Poisson regression was used to assess whether an increase in the number of mammograms and hospitalizations is related to the number of deaths, and the Cox–Stuart test was used to analyze the temporal trend of the variables under study and the projection of time series. Results: There was a strong positive correlation for all age groups when relating the variables hospitalizations and deaths, a moderate-to-strong correlation for the variables mammography and hospitalization, and a weak correlation for the variables mammography and death. There was no statistical significance in the relationship between the number of mammograms and deaths, whereas the hospitalization variable had a significant impact in relation to death, increasing the chance by 0.015%. There has also been a significant growth trend in the variables deaths and hospitalizations in Brazil over the years. Conclusions: A growing trend was identified from 2013 to 2021, both in hospitalizations and deaths, thus suggesting that strategies aimed at reformulating public health policies are necessary for earlier diagnosis in order to improve the treatment of breast cancer and the prognosis of the disease.

## 1. Introduction

Breast cancer ranks as the most commonly diagnosed cancer globally, according to data released by the International Agency for Research on Cancer [1] in 2020. In that year, breast cancer accounted for the highest incidence among all cancer types, comprising 47.8% of cases, and ranked as the second-leading cause of death, while also accounting for approximately 13.6% of all cancer-related deaths. The projected number of new cases worldwide for the year 2040 is 3.03 million. In European countries, the estimate stands at 568,439 cases, while in Latin America, it is 314,356 [2]. 

In Brazil, breast cancer claimed 17,825 lives in 2020, accounting for 2.61% of the total deaths attributed to cancer. The estimated number of new breast cancer cases for the three-year period from 2023 to 2025 is 73,610, representing an estimated risk of 66.54 new cases per 100,000 women. The highest estimated risk is observed in the southeast region, with 84.46 cases per 100,000 women, and in the central-west region, with 57.28 cases per 100,000 women [3].

Breast cancer holds a high likelihood of successful treatment when diagnosed early and managed effectively, requiring a comprehensive screening approach, with mammography examinations being the predominant method employed [4]. Recent research suggests that systematic screening of women deemed eligible (those within the age group at risk for breast cancer) through well-organized, high-quality programs can significantly reduce mortality associated with this disease. However, disparities exist in the implementation and organization of screening initiatives, impacting the coverage of women eligible for breast cancer screening [5].

According to the American Cancer Society’s Guidelines for Breast Cancer Screening, it is recommended that average-risk women aged 40–44 should begin screening with mammograms every year, women aged 45–54 should have mammograms every year, and women aged 55 and over, every two years, or opt for annual mammograms [6]. In Brazil, the Guidelines for the Early Detection of Breast Cancer recommend mammograms for women aged 50–69 every two years and consider mammography exams to be the only method proven effective for screening programs in both the early detection of breast cancer and reduced mortality [3].

Countries that adopted organized screening exams for the target group with biennial mammograms have been showing a reduction in breast cancer mortality [5]. In Brazil, breast cancer screening is carried out opportunistically. According to the author, digital image processing is an important point in the areas of research and development and is used to process digital images and generate useful characteristics from the data, which can then be used to make critical decisions with high precision [7]. Studies reveal a growing increase in the number of screening mammograms. However, there is low coverage among Brazilian states, a fact explained by the carrying out of opportunistic mammograms and those in women who do not belong to the target group as recommended by the Ministry of Health (50–69 years old), consequently reflecting the increase in the number of deaths annually [8]. Still, it is not clear whether there is an association between screening and diagnostic mammograms and the number of deaths. Some factors may be responsible for this divergence, such as the adoption of unorganized screening, socioeconomic and access inequality, and the exam not reaching the necessary coverage in the target population, thus making early diagnosis of the disease difficult and leading to an increase in the number of deaths from breast cancer. Studies have demonstrated the benefits of mammograms and improved survival [5,9,10], but there is no evidence of their correlation with hospitalizations and deaths.

Given this context, this study aims to analyze the association between the number of screening and diagnostic mammograms and the number of hospitalizations and deaths due to breast cancer.

## 2. Materials and Methods

### 2.1. Study Design

This is an observational, ecological study using secondary data available from the Brazilian Ministry of Health through the Computing Department of Brazil’s Unified Health System (DATASUS).

### 2.2. Data

The data collected were the number of diagnostics, screenings, and total mammograms carried out from 2013 to 2021, as well as hospitalizations and deaths resulting from breast cancer in Brazil among women aged 50–69 years.

### 2.3. Statistical Analysis

For data analysis, the R software version 4.1.0 was used (R Core Team, 2021). R: A language and environment for statistical computing. R Foundation for Statistical Computing, Vienna, Austria. URL https://www.R-project.org, accessed on 30 June 2024. The statistical methods applied in this study include the Pearson correlation test, Poisson regression model, Cox–Stuart test for trends, and time series projection using ARIMA models.

## 3. Results

Between 2013 and 2021, 11,782,041 mammograms were performed in general, of which 11,071,140 were screening mammograms and 710,901 were diagnostic mammograms. The years 2019, 2018, and 2017 were those with the highest number of mammograms performed in general, with 1,941,959 in 2019, 1,764,770 in 2018, and 1,671,219 in 2017. In 2020 and 2021, there was a drop in the overall number of mammograms, with 1,164,682 performed in 2020 and 1,667,657 in 2021. The average number of diagnostic mammograms in the period from 2013 to 2021 was highest in women aged 50–54, gradually reducing by the age of 69. The same occurred for screening mammograms and, consequently, for the total number of mammograms. The number of hospitalizations had the same trend within the age groups, presenting a higher average in the lower age group and gradually decreasing with higher ages. The number of deaths, on the other hand, had higher averages in the age groups between 55 and 64 years old.

There was a positive correlation for all age groups when comparing the variable hospitalizations × deaths, being classified as a very strong positive correlation for all age groups when added together; a strong positive correlation in the age groups of 55–59, 60–64, and 65–69; and a moderate positive correlation in the age group 50–54 (Table 1).

When analyzing the variable mammograms × deaths, the results vary in the age groups between weak and moderate positive correlations, with the highest value being *p* = 0.51 for all ages. The same occurred with the variable mammograms × hospitalizations, where the results vary between moderate and strong positive correlations, with the highest value found for all ages *p* = 0.60 (Table 1). There was a significant positive association between deaths due to breast cancer and the number of hospitalizations, and a protective factor when mammograms were realized (Table 2).

There has been a significant growth trend in the variables deaths and hospitalizations due to breast cancer in Brazil over the years; however, this has not occurred for any type of mammogram performed (diagnosis, screening, or total). No trend of growth or decrease was found in the number of mammograms performed during the study period (Table 3).

**Figure 1:**
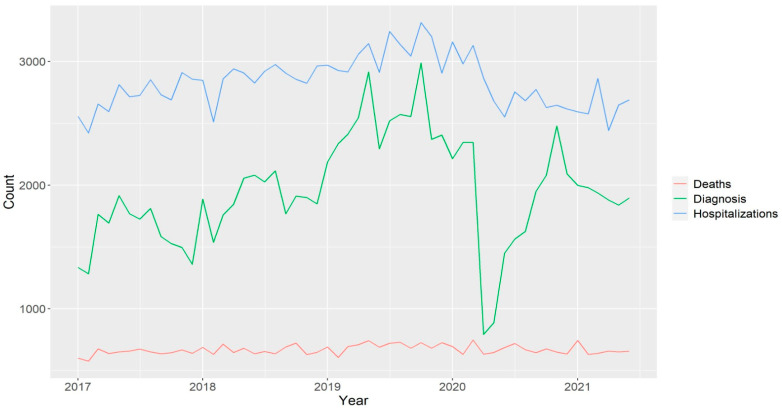
Time graph of the number of diagnostic mammograms, hospitalizations, and deaths due to breast cancer from January 2017 to May 2021 in Brazil.

It appears that, in the case of mammograms, a minimum peak occurred abruptly during the pandemic period, indicating that there was a considerable reduction in diagnostic mammograms performed. Regarding the number of hospitalizations, there had been an increasing trend over the years, followed by a decrease during periods when mammograms also reduced, soon resuming the previous magnitude (Figure 1).

**Figure 2:**
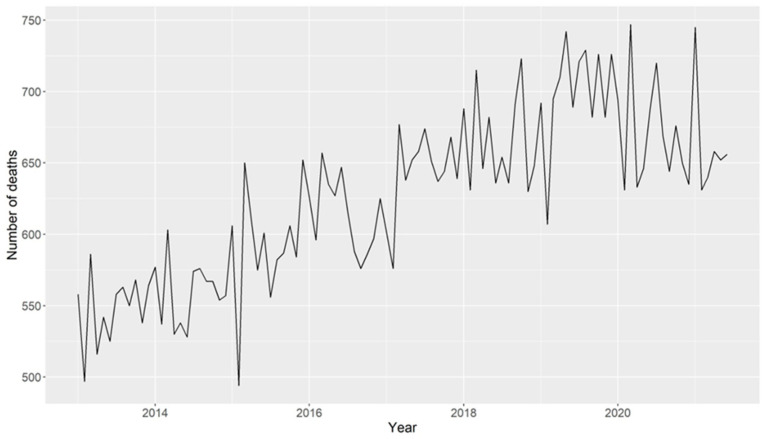
Time graph of deaths from breast cancer from 2013 to 2021 in Brazil.

Deaths from breast cancer did not have growth peaks or decreases as considerable as the other variables, showing a slight upward trend over the years (Figure 2). It is possible to verify that, despite the variations, there was a growth trend throughout the period, as indicated by the Cox–Stuart test.

## 4. Discussion

Breast cancer screening has an important effect on the diagnosis and treatment of breast cancer. In Brazil, studies reveal a growing increase in the number of mammograms for breast cancer screening and in the number of deaths from this disease, which contrasts with the reality in high-income countries, where the number of mammograms increases annually and the number of deaths has been showing a decrease [5,9,10,11].

The present study shows that screening mammograms comprise practically the entirety of exams carried out for this purpose in Brazil. However, regardless of the type of mammogram—screening or diagnostic—the trend was similar over time, differing only in the overall amount between both types of mammograms. Screening mammography for the early detection of breast cancer is still the most used method worldwide in asymptomatic women. It is considered the only imaging technique that has the proven benefit of significantly reducing mortality from breast cancer. A study carried out in Europe by Zielonke and colleagues (2021) [5] showed that mammography screening reduced the number of deaths from breast cancer by 34% across Europe. Similar data were found in the study by Ding and collaborators (2021) [12], also carried out in Europe, which shows that mammography screening programs reduced mortality from this disease by 25–30% in women with average risk in the age group 50–74 years old. In Norway, the reduction was 20% [13]. A Dutch study showed that the mammography invitational screening program reduced breast cancer mortality by 9.6% in women in the eligible age group [14].

The results of this study show that the correlation between hospitalizations and deaths tend to be stronger as the age range increases, suggesting a more solid relationship between these variables with aging. The correlations between mammograms and deaths and between mammograms and hospitalizations are also notable, especially in older age groups, indicating that mammograms may be associated with specific health outcomes at different ages. The relationship between hospitalizations and deaths from breast cancer in older women is a topic of great importance in the health sector. Studies have shown that the risk of developing breast cancer increases with advancing age [15]. However, it is critical to consider that mortality rates alone do not always reflect the intricacies of cancer, as factors such as access to medical care, stage of diagnosis, and treatment play crucial roles.

A recent study conducted by Smith et al. (2020) [16] analyzed a large sample of older women diagnosed with breast cancer and found that, although the rate of breast cancer–related hospitalizations increased with age, mortality did not follow the same trend. There are several factors that can influence this relationship. Older women with breast cancer may be receiving more effective treatments and adequate medical support, as well as the fact that age-specific hormonal and physiological factors can influence the behavior of the disease, positively reducing the death rate. It is important to highlight that the relationship between advanced age, hospitalizations, and deaths from breast cancer depends on several factors, including the patient’s general health, the presence of comorbidities, access to quality health services, and adherence to treatment. Therefore, personalized healthcare approaches are essential to improve the outcomes and quality of life for older women diagnosed with breast cancer.

During the period analyzed, there was no constant growing trend in the number of screening mammograms in Brazil. This could be justified by the COVID-19 pandemic, which began in 2020 and caused a reduction in both the supply of health services and the demand from women for this exam. Similar results were pointed out in other studies carried out in several countries, where there were decreases in the number of screening mammograms during the COVID-19 pandemic period, a consequence of social distancing, concerns about contagion, and a decrease in the population’s demand for medical care [17,18,19,20,21].

The number of breast cancer deaths in this study showed an increasing trend throughout the period. This result corroborates previous studies, which indicate that in the last 20 years, mortality rates from breast cancer have increased significantly. The incidence rate increased annually by 2.54% between 2006 and 2016, and the mortality rate increased by 12.2% between 1990 and 2015 [22]. The same trend can also be seen in China, in a study carried out by Sun et al. (2020) [23], which showed an increase in the mortality rate from 3.5% in 1990 to 10.5% in 2015. However, countries such as Estonia, Finland, Italy, and Portugal maintained a low mortality rate (30%) between 2011 and 2017, and countries such as Austria, Belgium, Bulgaria, Croatia, Czechia, Denmark, France, Germany, Iceland, Ireland, Lithuania, Netherlands, Norway, Spain, Sweden, Switzerland, and the United Kingdom had a decreasing trend in this same period, from 35% to 30%. Hungary and Latvia maintained a constant, high mortality rate (36%). On the other hand, Greece, Cyprus, Luxembourg, Poland, Romania, Serbia, Slovakia, and Slovenia all had an upward trend in the mortality rate from breast cancer, reaching 37% in 2017 [24].

One factor that may be responsible for this divergence in mortality rates between countries is the adoption of organized screening versus opportunistic screening strategies. Countries that use unorganized screening methods have greater difficulty achieving test coverage in the target population, thus making early diagnosis of the disease and, consequently, its prognosis more challenging. Examples are low and middle-income countries, where in addition to low coverage, there is insufficient capacity for laboratory diagnosis and barriers to treating the disease, such as inadequate treatment options and insufficient health information systems [25].

Results from international studies show that inviting women for breast cancer screening (organized screening) results in a 22% reduction in the mortality rate from the disease [26]. An example of this strategy’s validation is the United Kingdom, which carries out systematic, long-term prevention programs and promotes education effectively, thus maintaining a decrease in breast cancer mortality rates in recent years [24]. Attending screening exams also promotes a reduction in the risk of death from breast cancer by around 30% [26]. A meta-analysis study showed that organized screening carried out in countries such as Denmark, Italy, Spain, Sweden, and England promoted a significant reduction in the risk of death from breast cancer [27]. Since 2003, European countries have carried out organized breast cancer screening in accordance with the recommendations of the European Commission on the Breast Cancer Guideline Development Group (GDG) by inviting women between 45 and 49 years of age to undergo mammograms every 3 years and women between 50 and 74 years of age to undergo mammograms every 2 years [28].

## 5. Conclusions

This study showed that the number of screening mammograms was correlated with the number of hospitalizations for breast cancer (in all age groups), but there was no correlation with the number of deaths. However, diagnostic mammograms were correlated with both the number of hospitalizations and deaths (also for all age groups). One possible explanation for this difference is that, in regards to screening mammograms, the cancer is identified early, allowing death to be prevented. This does not necessarily occur in diagnostic mammograms, since the exam itself may already have been carried out by a motivation/body change in the patient. Furthermore, an increasing trend was identified from the years 2013–2021, both in hospitalizations and deaths from breast cancer. Mammograms had been increasing since 2017, but they underwent a sudden change during the pandemic period, drastically decreasing in number. From then on, screening mammograms soon resumed their previous trend, while diagnostic mammograms stabilized and have not shown an increasing trend since.

The limitation of this study comes from the lack of the most current data, since the Brazilian Ministry of Health’s Information System only provides mortality rates up to 2021, thus making it impossible to analyze the present health situation of the female population in the country. Nonetheless, the data collected are in accordance with the reality in countries that do not have organized screening.

Knowledge of the data on screening and deaths from breast cancer analyzed in this study is essential to provide information to society and the government in order to support better strategies for health policies, allowing for earlier diagnosis, better treatment, and better survival rates.

## Figures and Tables

**Table 1:** Pearson correlation tests between variables for each age group individually and combined, Brazil, 2023.

Test	Age Group	Correlation ( ρ)		
Hospitalizations × Deaths	50–54	0.50 *		
	55–59	0.67 *		
	60–64	0.66 *		
	65–69	0.74 *		
	All ages	0.82 *		
		Diagnostic	Screening	Total
Mammograms × Deaths	50–54	0.25	0.15	0.15
	55–59	0.34 *	0.06	0.06
	60–64	0.37 *	0.15	0.16
	65–69	0.47 *	0.21	0.22
	All ages	0.51 *	0.21	0.21
Mammograms × Hospitalizations	50–54	0.50 *	0.44 *	0.44 *
	55–59	0.49 *	0.46 *	0.46 *
	60–64	0.55 *	0.47 *	0.47 *
	65–69	0.60 *	0.57 *	0.58 *
	All ages	0.60 *	0.53 *	0.53 *

* Significant correlation at an α < 5%.

**Table 2:** Poisson regression model estimates between mammograms, hospitalizations, and deaths, Brazil, 2023.

	Estimate	Standard Error	Z Statistics	*p*-Value	Odds Ratio
Intercept	6.0773	0.0767	79.22	0.0000	435.87
Mammograms	−0.0000	0.0000	−0.73	0.4642	0.99
Hospitalizations	0.0001	0.0000	5.19	0.0000	1.00

**Table 3:** Cox–Stuart trend tests for the variables deaths, hospitalizations, and all types of mammography, Brazil, 2023.

Variable	Test Statistics	*p*-Value
Deaths	49	0.0000
Hospitalizations	48	0.0000
Diagnostic Mammograms	17	0.2326
Screening Mammograms	18	0.1366
Total Mammograms	18	0.1366

## Data Availability

The data presented in this study are publicly available.
